# Translational Development of a Zr-89-Labeled Inhibitor of Prostate-specific Membrane Antigen for PET Imaging in Prostate Cancer

**DOI:** 10.1007/s11307-021-01632-x

**Published:** 2021-08-09

**Authors:** Sergio Muñoz Vázquez, Heike Endepols, Thomas Fischer, Samir-Ghali Tawadros, Melanie Hohberg, Beate Zimmermanns, Felix Dietlein, Bernd Neumaier, Alexander Drzezga, Markus Dietlein, Klaus Schomäcker

**Affiliations:** 1 Faculty of Medicine and University Hospital Cologne, Department of Nuclear Medicine, University of Cologne, Kerpener Str. 62 50937, Cologne, Germany; 2 Faculty of Medicine and University Hospital Cologne, Institute of Radiochemistry and Experimental Molecular Imaging, University of Cologne, Kerpener Str. 62 50937, Cologne, Germany; 3 Forschungszentrum Jülich GmbH, Institute of Neuroscience and Medicine, Nuclear Chemistry (INM-5), Wilhelm-Johnen-Straße 52428, Jülich, Germany; 4 Faculty of Medicine and University Hospital Cologne, Center for Experimental Medicine (CEM), University of Cologne, Robert-Koch-Straße 10 50931, Cologne, Germany; 5 Department of Medical Oncology, Dana-Farber Cancer Institute, Harvard Medical School, Boston, MA USA

**Keywords:** Zirconium-89, Prostate carcinoma, Biochemical recurrence, PET imaging, Radiochemistry, Affinity, Cell uptake, Biokinetics

## Abstract

**Purpose:**

We present here a Zr-89-labeled inhibitor of prostate-specific membrane antigen (PSMA) as a complement to the already established F-18- or Ga-68-ligands.

**Procedures:**

The precursor PSMA-DFO (ABX) was used for Zr-89-labeling. This is not an antibody, but a peptide analogue of the precursor for the production of [^177^Lu]Lu-PSMA-617. The ligand [^89^Zr]Zr-PSMA-DFO was compared with [^68^Ga]Ga-PSMA-11 and [^18^F]F-JK-PSMA-7 *in vitro* by determination of the *K*_*d*_ value, cellular uptake, internalization in LNCaP cells, biodistribution studies with LNCaP prostate tumor xenografts in mice, and *in vivo* by small-animal PET imaging in LNCaP tumor mouse models. A first-in-human PET was performed with [^89^Zr]Zr-PSMA-DFO on a patient presenting with a biochemical recurrence after brachytherapy and an ambiguous intraprostatic finding with [^18^F]F-JK-PSMA-7 but histologically benign cells in a prostate biopsy 7 months previously.

**Results:**

[^89^Zr]Zr-PSMA-DFO was prepared with a radiochemical purity ≥ 99.9% and a very high *in vitro* stability for up to 7 days at 37 °C. All radiotracers showed similar specific cellular binding and internalization, *in vitro* and comparable tumor uptake in biodistribution experiments during the first 5 h. The [^89^Zr]Zr-PSMA-DFO achieved significantly higher tumor/background ratios in LNCaP tumor xenografts (tumor/blood: 309 ± 89, tumor/muscle: 450 ± 38) after 24 h than [^68^Ga]Ga-PSMA-11 (tumor/blood: 112 ± 57, tumor/muscle: 58 ± 36) or [^18^F]F-JK-PSMA-7 (tumor/blood: 175 ± 30, tumor/muscle: 114 ± 14) after 4 h (*p* < 0.01). Small-animal PET imaging demonstrated *in vivo* that tumor visualization with [^89^Zr]Zr-PSMA-DFO is comparable to [^68^Ga]Ga-PSMA-11 or [^18^F]F-JK-PSMA-7 at early time points (1 h p.i.) and that PET scans up to 48 h p.i. clearly visualized the tumor at late time points. A late [^89^Zr]Zr-PSMA-DFO PET scan on a patient with biochemical recurrence (BCR) had demonstrated intensive tracer accumulation in the right (SUV_max_ 13.25, 48 h p.i.) and in the left prostate lobe (SUV max 9.47), a repeat biopsy revealed cancer cells on both sides.

**Conclusion:**

[^89^Zr]Zr-PSMA-DFO is a promising PSMA PET tracer for detection of tumor areas with lower PSMA expression and thus warrants further clinical evaluation.

**Supplementary Information:**

The online version contains supplementary material available at 10.1007/s11307-021-01632-x.

## Introduction

In current clinical practice, tumor localization in patients with biochemical recurrence (BCR) of prostate cancer is the most accepted and validated field of application of PET/CT with Ga-68 or F-18- prostate-specific membrane antigen (PSMA) ligands [1–3]. However, in an estimated 20% of patients with biochemical recurrence the tumor will remain undetected with conventional PSMA PET imaging approaches [4–6].

There are two main reasons for this: First, about 5–10% of primary prostate cancers express no PSMA [7, 8]. Second, different tumors can exhibit a marked heterogeneity in the proportion of PSMA-positive cells they contain [9–11].

It cannot be ruled out that weakly PSMA-expressing prostate carcinoma foci are overlooked when using short-lived radionuclides for a PET scan [12]. A negative finding after PSMA PET/CT examinations using Ga-68- or F-18-ligands therefore represents a selection of prostate carcinoma foci with absent or weak PSMA expression. Our aim was to develop a PSMA ligand that can localize BCRs with weak PSMA expression on the basis of late acquisition windows (2 days after injection or later) and hence achieve an improved lesion-to-background ratio. The intention of this study was not to develop an alternative to short-lived radiotracers, but to expand the range of available PET tracers if, despite rising prostate-specific antigen (PSA) values, no convincing tumor detection with Ga-68 or F-18 PSMA tracers is possible. We designed [^89^Zr]Zr-PSMA-DFO for the rare constellation of a BCR, a preceding PSMA-negative scan with Ga-68- or F-18-PSMA ligands, and the preference for metastasis-directed therapy over androgen deprivation therapy.

To this end, we investigated a new PSMA-binding compound that exploits the longer physical half-life (78.41 h) of Zr-89: [^89^Zr]Zr-*N*-*suc*Df-AMCHA-2Nal-EuK (*N*-sucDf: *N*-succineimidedesferrioxamine, AMCHA: tranexamic acid, 2Nal: 2-naphthyl-alanine, E: glutamic acid, u: urea, K: lysine; [^89^Zr]Zr-PSMA-DFO). The ligand itself is an analogue of the precursor used to prepare [^177^Lu]Lu-PSMA-617. The only difference lies in the chelating agent for zirconium binding. While in DFO-PSMA, the chelating agent is *N*-sucDf (blocked temporarily with Fe(III)), in PSMA-617 DOTA is used for this purpose. So in this study, it is not an antibody that was used for Zr-89 labeling but a small molecule.

Previous studies with Zr-89 have focused primarily on antibody-based PET imaging [13, 14], as the physical half-life of this radionuclide fits well with the biological half-life of the commonly used antibody constructs. The radionuclide Zr-89 decays into the stable isotope Y-89 by positron emission (23%) and electron capture (77%). The *E*_max_ of 897 keV and the *E*_ave_ of 396.9 keV of the emitted positrons are low enough to produce PET images of good spatial resolution. The possibly distorting, spontaneous gamma decay of Zr-89 with 908.97 keV photons (99% abundance) can be masked by adjusting the energy window of the PET scanner [15]. The radionuclide has not previously been used to target PSMA-expressing prostate cancer lesions.

Our first aim with this study was to show that the radiotracer can be produced in good radiochemical quality.

**Fig. 1:**
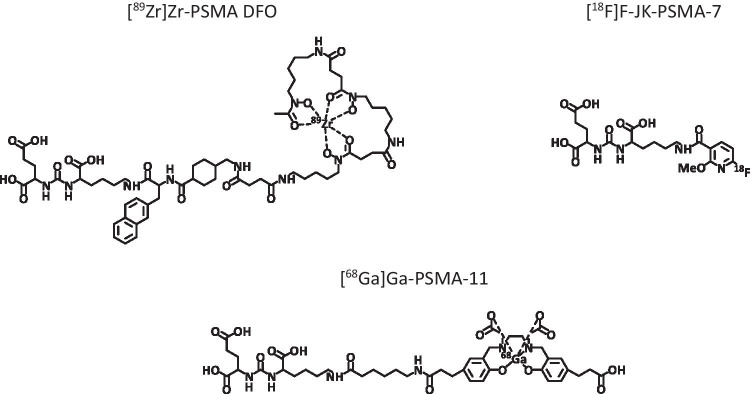
Structural formulas of the radioligands examined. [^68^Ga]Ga-PSMA-11 and [^89^Zr]Zr-PSMA-DFO contain 3 components: the pharmacophore lysine-urea-glutamate, the chelating agent, and the linker. For [^68^Ga]Ga-PSMA-11, HBED-CC and 6-aminohexanoic (ahx) were used as chelator and linker, respectively. For [^89^Zr]Zr-PSMA-DFO, DFO was used as chelator and naphthylalanine and tranexamic acid (2NaI-AMCHA) were functionalized as linker. In the case of [^18^F]F-JK-PSMA-7, the lysine-urea-glutamate scaffold was coupled to tetrafluorophenyl 6-fluoro-4-methoxynicotinate (6-F-4-OMe-Nic-OTfp).

The second aim, carried out in the cell biological/biochemical part of the work, was to compare the [^89^Zr]Zr-PSMA-DFO vector with short-lived radiotracers targeting PSMA ([^68^Ga]Ga-PSMA-11, [^18^F]F-JK-PSMA-7 [5, 16–18]) with regard to stability, affinity, cell uptake, and internalization in PSMA-positive cells. The structural formulas of the examined radioligands are shown in Fig. 1.

Third, the animal experimental part of the study was designed to test the hypothesis that higher tumor-to-background ratios can be achieved with [^89^Zr]Zr-PSMA-DFO than with the short-lived tracers.

Fourth, [^89^Zr]Zr-PSMA-DFO was to be applied for the first time in a selected patient with BCR as part of the clinical workup.

The key question was whether the results achievable with [^89^Zr]Zr-PSMA-DFO are at least equal or better to those achieved with [^68^Ga]Ga-PSMA-11 and [^18^F]F-JK-PSMA-7.

## Materials and Methods

PSMA-11, EuK-2Nal-AMCH-N-sucDf-Fe, and disposable cassette kits were produced by ABX (Radeberg, Germany). Water (Ultrapur^﻿®^), hydrochloric acid 30% (Ultrapur^﻿®^), and sodium hydroxide 30% (Suprapur^﻿®^) were obtained from Merck Millipore (Steinheim, Germany). Serum was collected from whole blood in serum separator tubes. Zr-89 in 1 M oxalic acid was supplied by Perkin Elmer and produced by BV Cyclotron VU (Amsterdam, Netherland). Ga-68 was eluted in a solution of 0.1 M HCl from a Ge-68/Ga-68-Generator Eckert & Ziegler Radiopharma (Berlin, Germany). The other chemicals used were acquired from Sigma-Aldrich (Darmstadt, Germany).

### Radiolabeling and Complex-Stability Studies

[^68^Ga]Ga-PSMA-11 and [^18^F]F-JK-PSMA-7 were labeled according to standard protocols, which have been published elsewhere [18].

The precursor EuK-2NaI-AMCHA-N-sucDf-Fe used for the Zr-89-based PSMA-vector was formed by coupling the pharmacophore EuK to a naphthylic linker and the chelator agent *N*-sucDf-Fe. The *N*-sucDf-Fe moiety functionalized the molecule for labeling with Zr-89. It proved to be a suitable chelator for [^89^Zr]-zirconium [19].

Labeling of the precursor Fe–N-PSMA-DFO with Zr-89 required a multistep procedure due to the presence of Fe(III).

After preparation of the iron-free PSMA-DFO, the radiolabeling procedure was performed by adjusting the pH of a solution of Zr-89 (20–600 MBq) in 1 M oxalic acid to 6.8–7.2 with 1 M sodium carbonate, 0.5 M HEPES (pH 6.8), and 0.25 M sodium acetate (5 mg/1 ml gentisic acid, 50 µl). Once the desired pH had been reached, a known amount of PSMA-DFO (5, 10, or 20 nmol) was added and the reaction was monitored at different incubation times. The sets of results produced by changing the reaction parameters, such as pH, amount of precursor, and incubation time, were evaluated to determine the highest radiochemical yield. The unbound Zr-89 was then efficiently removed by solid-phase extraction using a Sep-Pak™ C_18_ plus light cartridge.

A complete description of the Zr-89 labeling procedure can be found in the Supplementary information as Supplementary Fig. 1.

To test the release of low molecular weight zirconium species from the PSMA-targeting radiotracer, 100 μl of [^89^Zr]Zr-PSMA-DFO were added to either 1 ml PBS (PAA Laboratories, Pasching Austria) or 1 ml human serum. The solutions were kept at a constant temperature of 37 °C by means of a heating block (Dry Block Heater 1, IKA, Staufen, Germany) for different periods of time after thorough mixing. The samples were checked for radiochemical purity by radio-thin-layer chromatography immediately after reaching a temperature of 37 °C and again after periods of 1 h, 2 h, 24 h, 48 h, and 72 h. The [^89^Zr]Zr-PSMA-DFO remained at the origin of the salicylic acid impregnated instant thin-layer chromatography (ITLC) strip (Agilent, CA, USA), while free Zr-89 migrated with the mobile phase (citrate buffer of 0.5 M pH 5.0). The stability of the complexes was calculated as the percentage of complexes remaining at the origin. The radiochemical purity was additionally determined by HPLC (Column: Macherey–Nagel, NUCLEODUR C_18_ gravity 5 µm, 110 Å, 250 × 4 mm) at a flow rate of 1.2 ml/min. Elution with 2 min 5% B was followed by the beginning of the gradient of 5–95% B in 15 min, followed by a hold of 5 min. The A solvent was 0.1% trifluoroacetic acid in water, while 0.1% trifluoroacetic acid in acetonitrile was the B solvent. The unreacted Zr-89 was eluted at retention time *R*_*t*_ = 2.01 min, while the [^89^Zr]Zr-PSMA-DFO had a *R*_*t*_ = 9.36 min.

### Equilibrium Dissociation Constant* K*_*d*_

The equilibrium dissociation constant describing the interaction of the radiolabeled ligands with the PSMA binding sites was determined in PSMA-positive LNCaP cell lines (Cell Line Service, Eppelheim, Germany). LNCaP cells were seeded at a density of 10^6^ cells/well on a 6-well plate (Corning, ME, USA) and incubated under 5% CO_2_ at 37 °C for 48 h with medium (minimum essential medium Eagle supplemented with 2 mM l-glutamine, 0.1 mM non-essential amino acids (NEAA), 1.0 mM sodium pyruvate, 10% fetal bovine serum, 100 U/ml penicillin, and 100 µg/ml streptomycin) (Lonza, Verviers, Belgium) in a humidified incubator (Thermo Heracell 150 CO_2_-Incubator, MA, USA). To determine the nonspecific binding, the medium was removed from selected wells, and 1 ml of a solution of 0.1 mM of 2-(phosphonomethyl)-pentanedioic acid (2-PMPA) in fresh medium was added. 2-PMPA reliably blocks the PSMA binding sites [20, 21]. The plates were then incubated under 5% CO_2_, at 37 °C for 1 h. After that, the medium was removed and the cells incubated under the same incubation conditions for 3 h with different concentrations (0.25 nM, 2.5 nM, 5 nM, 10 nM, 25 nM, 50 nM, and 75 nM) of the radiotracer under investigation in 1 ml fresh medium.

After 3 h, the cells were washed three times with 1 ml PBS, and the cells were finally lysed by adding 1 ml of 1 M NaOH and incubating them for 10 min at room temperature. All samples were measured with a gamma counter (Nuklear Medizintechnik Dresden Isomed 100, Dresden, Germany) and were decay-corrected. The equilibrium dissociation constant (*K*_*d*_) and the maximum density of receptors (*B*_max_) were calculated by non-linear regression using GraphPad Prism 8.0.2 (GraphPad Software, San Diego, USA).

### Cellular Uptake and Internalization

The cell binding determined in these experiments is the sum of specific and nonspecific cell binding. To determine the values for nonspecific cell binding and internalization, all experiments were additionally performed in the presence of 2-PMPA.

The plates were incubated for 1 h; after which, 0.75 pmol of the radiotracer to be examined was added to 1 ml of fresh medium. The cells were incubated for 30 min, 1, 2, 3, and 5 h. At each time point, the supernatant was removed and the cells washed with 1 ml of PBS. To dissociate the receptor-bound radioligand, the cells were washed twice with 1 ml of a 0.1 M glycine buffer solution at pH 2.8 for 5 min. The 0.1 M glycine–HCl buffer dissociated all the surface-bound complexes. The cells were then washed with 1 ml of PBS, and the internalized fraction was determined by solubilizing the cells with 1 ml of 1 M NaOH and incubating them for 10 min at room temperature. The radioactivity collected from the culture medium, 0.1 M glycine (surface-bound), and 1 M NaOH (internalized fraction) was measured in a gamma counter and decay-corrected. Cell binding was calculated from the surface-bound (0.1 M glycine) and the internalized fraction (1 M NaOH). The internalized fraction was expressed as a percentage of cell binding (internalization to cell-bound radioactivity ratio). All cell uptake experiments were run in triplicate.

### Biodistribution in Animal Tissue

After application of the various radiotracers, these experiments were used to determine the radioactivity accumulation in various tissues (percent of the applied radioactivity per gram of organ) and then to calculate tumor/blood, tumor/muscle, tumor/liver, and tumor/kidney ratios.

Animal experiments were performed in strict accordance with the European Union directive 2010/60/EU for animal experiments, and with the approval of the regional authorities (Ministry for Environment, North Rhine-Westphalia).

A total of 35 mice were used for biodistribution experiments. Male CB17-SCID mice (age: 6 weeks, weight: 17–20 g) were purchased from Charles River Laboratories (Wilmington, USA). Mice were kept in groups of 3–5 with free access to water and food in individually ventilated cages (NexGen EcoFlo, cages Mouse500; Allentown Inc., Allentown, NJ, USA) under controlled conditions (22 ± 1 °C and 55 ± 5% rh) and a 12-h light/dark schedule. The day before implantation of the LNCaP cells, 20 µl of Anti-Asialo GM1 Rabbit (1 mg ml^−1^ 0.9% NaCl) (FUJIFILM Wako Chemicals GmbH, Neuss, Germany) was injected into each mouse. This was done in order to transiently suppress natural killer cell activity, which is preserved in SCID mice [22]. Tumor cells for implantation were harvested by trypsination (TrypLE™ Express, Life Technologies, Paisley, UK) and 8.7 × 10^6^ cells in 150 µl PBS with Ca^2+^/Mg^2+^ 1:1 with Corning^﻿®^ Matrigel^﻿®^ matrix (Corning, NY, USA) were inoculated subcutaneously into the right flank of each mouse. After inoculation, the mice were monitored periodically until the cells had formed a tumor of 300 to 600 mg (approximately 6 weeks). The mice were then used either for biodistribution studies or for PET imaging.

On the day of the experiment, each animal was injected intravenously via the tail vein with 30 pmol of [^89^Zr]Zr-PSMA-DFO (approximately 1 MBq/100 µl) or [^18^F]F-JK-PSMA-7 (1 MBq/100 µl), or [^68^ Ga]Ga-PSMA-11 (1 MBq/100 µl). Five mice were selected for each time point and type of PSMA-targeting radiotracer. The mice were sacrificed by cervical dislocation at 2 and 4 h after injection of [^18^F]F-JK-PSMA-7 or [^68^Ga]Ga-PSMA-11 and at 2, 4 and 24 h after administration of [^89^Zr]Zr-PSMA-DFO.

The organs to be studied (blood, liver, spleen, kidneys, muscle, bone, thyroid, lungs, intestines, tumor, heart, and prostate) were dissected out and weighed. The radioactivity in samples was counted in a gamma counter and decay-corrected. The results for each labeled urea-based inhibitor are expressed as a percent of the injected dose per gram of tissue (% ID/g) and presented as means ± standard deviations (SD) (*n* = 5).

### Small-Animal PET in Mice Bearing an LNCaP Tumor Xenograft

PET imaging was used to confirm the results of the biodistribution experiments. [^68^Ga]Ga-PSMA-11 and [^18^F]F-JK-PSMA-7 PET (10 MBq of the respective tracer per animal) were acquired for descriptive purposes only. Hence, only one mouse per radiotracer was used for small-animal PET comparisons. The binding specificity of [^89^Zr]Zr-PSMA-DFO was tested using the PSMA blocker 2-PMPA (23 mg/kg, *n* = 3 with and *n* = 3 without 2-PMPA). Scans were conducted under anesthesia on a Focus 220 micro-PET scanner (CTI-Siemens, Germany). Prior to PET imaging, the animals were anesthetized by inhalation of 5% isoflurane/gas mixture (O_2_/air 3:7). Thereafter, the anesthesia was reduced and maintained at a concentration of 2% isoflurane/gas mixture.

Emission scans were performed for 60 min, starting 60 min after tracer injection. Additional scans of 60 min duration were performed 4 h, 21 h, and 48 h after injection of [^89^Zr]Zr-PSMA-DFO. All emission scans were followed by a 10-min transmission scan with a Co-57 point source for attenuation correction. Summed images were reconstructed using an iterative OSEM3D/MAP procedure resulting in voxel sizes of 0.47 × 0.47 × 0.80 mm. Post-processing and image analysis was performed with VINCI 4.72 (Max-Planck-Institute for Metabolism Research, Cologne, Germany). Images were Gauss-Filtered (1 mm FWHM) and intensity-normalized to injected dose, corrected for body weight (SUV_bw_). For this, every frame was divided by injected dose and multiplied by 100 * body weight.

### Patient PET/CT Scan

A first-in-human study with [^89^Zr]Zr-PSMA-DFO was performed on a 60-year-old patient with BCR (PSA 3.2 ng/ml, nadir 0.66 ng/ml) as part of the clinical workup. The patient had undergone brachytherapy of the prostate cancer and displayed an increase in PSA level. Seven months previously, [^18^F]F-JK-PSMA-7 PET/CT had shown a weak PSMA-positive intraprostatic finding, but a prostate biopsy had contained only histologically benign cells. Findings from a repeat [^18^F]F-JK-PSMA-7 PET/CT 7 months later were again ambiguous in the right and left prostate lobe dorsal. After the BCR had failed to be localized by the repeat [^18^F]F-JK-PSMA-7 PET/CT, an additional [^89^Zr]Zr-PSMA-DFO PET was recommended as an individual clinical indication. The patient had given his written informed consent for PET imaging and the scientific evaluation of his data. All procedures were performed in accordance with the Institutional Review Board and the regulations of the regional authorities in Cologne. The kidney dose was estimated on the basis of two PET scans. The following assumptions were made for the estimation: Between time 0 (injection) and the first measuring point, the time-activity-curve follows a constant progression. All measuring points were integrated numerically using trapezoidal approximation. From the last measuring point to infinity, a mono-exponential function was fitted and integrated. As the effective half-life could not be accurately determined from two measurement points, the physical half-life of Zr-89 was used instead. Regarding the noise in the [^89^Zr]Zr-PSMA-DFO scans (93 MBq [^89^Zr]Zr-PSMA-DFO versus 343 MBq [^18^F]F-JK-PSMA-7), we measured the signal-to-noise ratio (SNR) for all PET/CT scans and the contrast-to-noise ratio (CNR) for the [^89^Zr]Zr-PSMA-DFO-avid lesions and the corresponding areas in the [^18^F]F-JK-PSMA-7 PET.

### Statistical Analysis

Statistical analyses were performed using GraphPad Prism 8.0.2 (version 8.0.2 for Windows GraphPad Software, San Diego, USA). For the cell uptake experiments, a 2-way ANOVA was performed with the factors radioligand and time point. For the biodistribution experiments, two separate 3-way ANOVA tests were done ([^89^Zr]Zr-PSMA-DFO vs. [^68^Ga]Ga-PSMA-11 and [^89^Zr]Zr-PSMA-DFO vs. [^18^F]F-JK-PSMA-7, respectively) with the factors organ, radioligand, and time point. As the 24 h values were available for [^89^Zr]Zr-PSMA-DFO only, this time point was not included in the 3-way ANOVA. To compare the [^89^Zr]Zr-PSMA-DFO 24 h p.i. to the other tracers and other times p.i., a mixed-effects analysis was used for organ uptake, and 1-way ANOVAs for the tumor-to-blood, -kidney- and -muscle ratios. For the PET experiments with the tumor xenograft–bearing mice, three separate 2-way mixed design ANOVA tests (for tumor, liver, and kidneys, respectively) were used with the factors blocking ([^89^Zr]Zr-PSMA-DFO with or without the blocking agent 2-PMPA) and time point (repeated measures). All ANOVA tests were followed by Sidak’s or Tukey’s multiple comparison procedures. The significance level was always *p* < 0.05.

## Results

### Radiochemistry and Stability

After the removal of free Zr-89 by solid-phase extraction using a Sep-Pak™ C_18_ plus light cartridge, the radiochemical purity of [^89^Zr]Zr-PSMA-DFO was ≥ 99.9%. The yield of [^89^Zr]Zr-PSMA-DFO in the radiolabeling method was 60.72 ± 5.50%. The molar activity (*A*_*m*_) reached was 60 MBq/nmol. For comparison, the molar activities of the radioligands used in the following experiments are shown in Table 1.

**Table 1:** Molar activities (*A*_*m*_) of the radioligands used in the experiments

Radioligand	A_m_ [MBq/nmol]
[^89^Zr]Zr-PSMA-DFO	60.4 ± 5.1
[^68^Ga]Ga-PSMA-11	71.0 ± 9.3
[^18^F]F-JK-PSMA-7	110 ± 15.2

The Zr-89-radioligand was found to be stable over a period of 7 days at 37 °C in PBS and human serum. The stability test was performed in thin-layer chromatography (TLC) solely to identify free Zr-89 at 1 h, 2 h, 24 h, 48 h, 72 h, and 7 days. However, the stability in PBS was measured in parallel with HPLC at same time intervals, and one single peak was identified at the retention time of [^89^Zr]Zr-PSMA-DFO (*R*_*t*_ = 9.35 min).

### Affinity, Cell Binding, and Internalization of [^89^Zr]Zr-PSMA-DFO in Comparison with [^18^F]F-JK-PSMA-7 and [^68^Ga]Ga-PSMA-11

The radioligands showed no important differences with regard to their *in vitro* behavior when interacting with the LNCaP cells. The binding curves including Scatchard Plots are shown in Supplementary Fig. 2. Table 2 gives an overview of the results of these investigations.

**Table 2:** Results of investigations on radioligand binding to PSMA-positive LNCaP cells based on Scatchard plots

Radiolabeled ligand	*K*_d_^*^ [nM]	*B*_max_^**^ [fmol/10^6^ cells]
[^89^Zr]Zr-PSMA-DFO	4.97 ± 0.57	1428 ± 42
[^68^Ga]Ga-PSMA-11	5.15 ± 0.60	1746 ± 53
[^18^F]F-JK-PSMA-7	5.07 ± 0.45	2702 ± 61

^*^K_*d*_ Dissociation constant in nmol

^**^B_*max*_ Maximum achievable concentration on 10^6^ tumor cells

Checking the relationship between *B*_max_ and molar activity by linear regression reveals a clear linear relationship: *B*_max_ = 25.37 *A*_*m*_—82.63 (*R*^2^ = 0.9986, *p* = 0.002).

The specific binding of radioactively labeled ligands to LNCaP cells, as a percentage of the total activity, ranged between 45.8 and 49.4% after 5 h. The specific internalized activity in LNCaP cells, expressed as a percentage of the cell activity relative to the specific cell-bound activity, was between 58 and 62% after 5 h. More detailed information on the respective values and their statistics can be found in the Supplementary information (Supplementary Tables 1 and 2).

### Biodistribution in Animal Tissue

A 3-way ANOVA was performed to compare organ uptake of [^89^Zr]Zr-PSMA-DFO vs. [^68^Ga]Ga-PSMA-11 and of [^89^Zr]Zr-PSMA-DFO vs. [^18^F]F-JK-PSMA-7 (Table 3). Main effects for factor tracer were *F*(1,119) = 161.1; *p* < 0.0001 and *F*(1,12) = 48.0; *p* < 0.0001, respectively, indicating that tracers differed with respect to their biodistribution. Tukey’s multiple comparison revealed that the uptake of [^89^Zr]Zr-PSMA-DFO into the LNCaP tumor xenograft was comparable to that of [^68^Ga]Ga-PSMA-11 and [^18^F]F-JK-PSMA-7 after 2 h and 4 h. Three ratios were calculated (tumor/blood, tumor/kidney, and tumor/muscle), and a 2-way ANOVA comparison performed on each. Tumor/blood ratios (*F*(2,18) = 26.0; *p* < 0.0001 for factor tracer) were the highest for [^89^Zr]Zr-PSMA-DFO, and significantly different from [^68^Ga]Ga-PSMA-11 at both time points. After 24 h, [^89^Zr]Zr-PSMA-DFO reached the exceptionally high tumor/blood ratio of 309 ± 89 (Table 3). This was confirmed by a one-way ANOVA, where significant differences were found compared to [^68^ Ga]Ga-PSMA-11 and [^18^F]F-JK-PSMA-7 with the time points 2 h an 4 h (*F*(6,20) = 16.9, *p* < 0.0001, post hoc* p* < 0.05).

**Table 3:** Biodistribution data (%ID/g) of [^89^Zr]Zr-PSMA-DFO, [^68^Ga]Ga-PSMA-11, and [^18^F]F-JK-PSMA-7 in CB17-SCID mice bearing LNCaP tumor xenografts (*n* = 5) for each tracer and time point)

	[^89^Zr]Zr-PSMA-DFO			[^68^Ga]Ga-PSMA-11		[^18^F]F-JK-PSMA-7	
	2 h	4 h	24 h	2 h	4 h	2 h	4 h
LNCAP-tumor	26.3 ± 5.3	22.8 ± 1.5	14.9 ± 1.2	19.4 ± 2.4	23.2 ± 10.6	20.2 ± 2.4	21.3 ± 1.5
Liver	0.95 ± 0.15	0.79 ± 0.17	0.37 ± 0.03	3.17 ± 0.18**	0.65 ± 0.21	4.44 ± .0.78*	3.75 ± 0.50**
Spleen	0.49 ± 0.07	0.61 ± 0.09	0.36 ± 0.08	40.9 ± 9.3**^§^	62.5 ± 21.5**^§^	2.15 ± 0.45*	1.57 ± 0.22*
Kidneys	86.7 ± 18.7^§^	41.3 ± 2.37^§^	15.4 ± 4.1	231 ± 41*^§^	226 ± 25^§^	27.2 ± 2.6	26.0 ± 3.5*
Blood	0.14 ± 0.02	0.13 ± 0.04	0.05 ± 0.01	1.34 ± 0.20	0.21 ± 0.03	0.15 ± 0.04	0.12 ± 0.03
Muscle	0.05 ± 0.02	0.08 ± 0.02	0.03 ± 0.01	0.54 ± 0.15	0.42 ± 0.11	0.07 ± 0.02	0.19 ± 0.03
Bone	0.15 ± 0.04	0.13 ± 0.03	0.18 ± 0.02	1.19 ± 0.31	0.50 ± 0.19	0.18 ± 0.03	0.20 ± 0.03
Thyroid	0.18 ± 0.03	0.13 ± 0.02	0.05 ± 0.01	2.13 ± 0.27*	4.44 ± 2.9	0.19 ± 0.02	0.20 ± 0.02
Lung	0.5 ± 0.04	0.12 ± 0.03	0.04 ± 0.01	3.27 ± 1.03	1.23 ± 0.13*	0.18 ± 0.01	0.18 ± 0.04
Intestine	0.65 ± 0.15	0.54 ± 0.10	0.25 ± 0.08	1.50 ± 0.59	0.65 ± 0.30	0.89 ± 0.18	1.53 ± 0.31
Heart	0.11 ± 0.05	0.12 ± 0.03	0.09 ± 0.02	1.10 ± 0.36	1.35 ± 0.70	0.12 ± 0.02	0.10 ± 0.02
Prostate	0.15 ± 0.04	1.12 ± 0.16	0.50 ± 0.01	4.09 ± 1.15	1.67 ± 0.37	0.22 ± 0.03	0.60 ± 0.16
Tumor/blood	183 ± 42^#^	180 ± 61^#^	309 ± 89	15 ± 3*^§^	112 ± 57*^§^	142 ± 24^§^	175 ± 30^§^
Tumor/kidneys	0.31 ± 0.06^§^	0.55 ± 0.01^§^	1.02 ± 0.28	0.09 ± 0.02**^§^	0.10 ± 0.05**^§^	0.74 ± 0.08**	0.83 ± 0.13**
Tumor/muscle	534 ± 62	297 ± 62^§^	450 ± 38	37 ± 8**^§^	58 ± 36**^§^	282 ± 71**^§^	114 ± 14**^§^
Tumor/liver	27.5 ± 3.2^§^	29.6 ± 5.1^§^	40.0 ± 1.1	6.2 ± 1.0**^§^	34.6 ± 6.6	4.7 ± 1.2**^§^	5.7 ± 0.7**^§^

^*^p < 0.05, **p < 0.01; significantly different from [^89^Zr]Zr-PSMA-DFO uptake at the corresponding time point (three-way ANOVA followed by Tukey’s multiple comparisons test for organs, two-way ANOVA for ratios)

^#^*p* < 0.05, ^§^*p* < 0.01; significantly different from [^89^Zr]Zr-PSMA-DFO uptake at 24 h (two-way ANOVA, mixed-effects model, followed by Tukey’s multiple comparisons test for organs, one-way ANOVA for ratios)

Tumor/kidney ratios (*F*(2,18) = 172.7; *p* < 0.0001) were the highest for [^18^F]F-JK-PSMA-7 after 2 h and 4 h, and significantly higher compared to [^89^Zr]Zr-PSMA-DFO, while [^68^Ga]Ga-PSMA-11 tumor/kidney ratios were significantly lower. After 24 h, however, the tumor/kidney ratio of [^89^Zr]Zr-PSMA-DFO (1.0 ± 0.3) surpassed the highest value of [^18^F]F-JK-PSMA-7 (0.83 ± 0.13). One-way ANOVA revealed that the tumor/kidney ratio of [^89^Zr]Zr-PSMA-DFO was significantly higher after 24 h than at earlier time points, and compared to [^68^Ga]Ga-PSMA-11 at 2 h and 4 h (*F*(6,20) = 38.3, *p* < 0.000, post hoc* p* < 0.05).

Tumor/muscle ratios (*F*(2,18) = 102.7; *p* < 0.0001) were highest for [^89^Zr]Zr-PSMA-DFO, and significantly different from [^68^Ga]Ga-PSMA-11 and [^18^F]F-JK-PSMA-7 at 2 h and 4 h. After 24 h, tumor/muscle ratio was still 450 ± 38 for [^89^Zr]Zr-PSMA-DFO. One-way ANOVA showed that this was significantly higher compared to [^68^Ga]Ga-PSMA-11 and [^18^F]F-JK-PSMA-7 at 2 h and 4 h (*F*(6,20) = 62.1, *p* < 0.0001, post hoc* p* < 0.05).

Tumor/liver ratios (*F*(2,10) = 101.7; *p* < 0.0001) of [^89^Zr]Zr-PSMA-DFO were significantly higher compared to [^68^Ga]Ga-PSMA-11 and [^18^F]F-JK-PSMA-7 at 2 h, and significantly higher compared to [^18^F]F-JK-PSMA-7 at 4 h. Tumor/liver ratio of [^68^Ga]Ga-PSMA-11 at 4 h was comparable to that of [^89^Zr]Zr-PSMA-DFO. After 24 h, tumor/liver ratio of [^89^Zr]-PSMA-DFO was significantly higher than after 2 h and 4 h, and significantly higher than tumor/liver ratio of [^68^Ga]Ga-PSMA-11 and [^18^F]F-JK-PSMA-7 at 2 h and [^18^F]F-JK-PSMA-7 at 4 h (*F*(6,20) = 102.3; *p* < 0.0001, post hoc* p* < 0.05).

### Small-Animal PET in Mice Bearing an LNCaP Tumor Xenograft

**Fig. 2:**
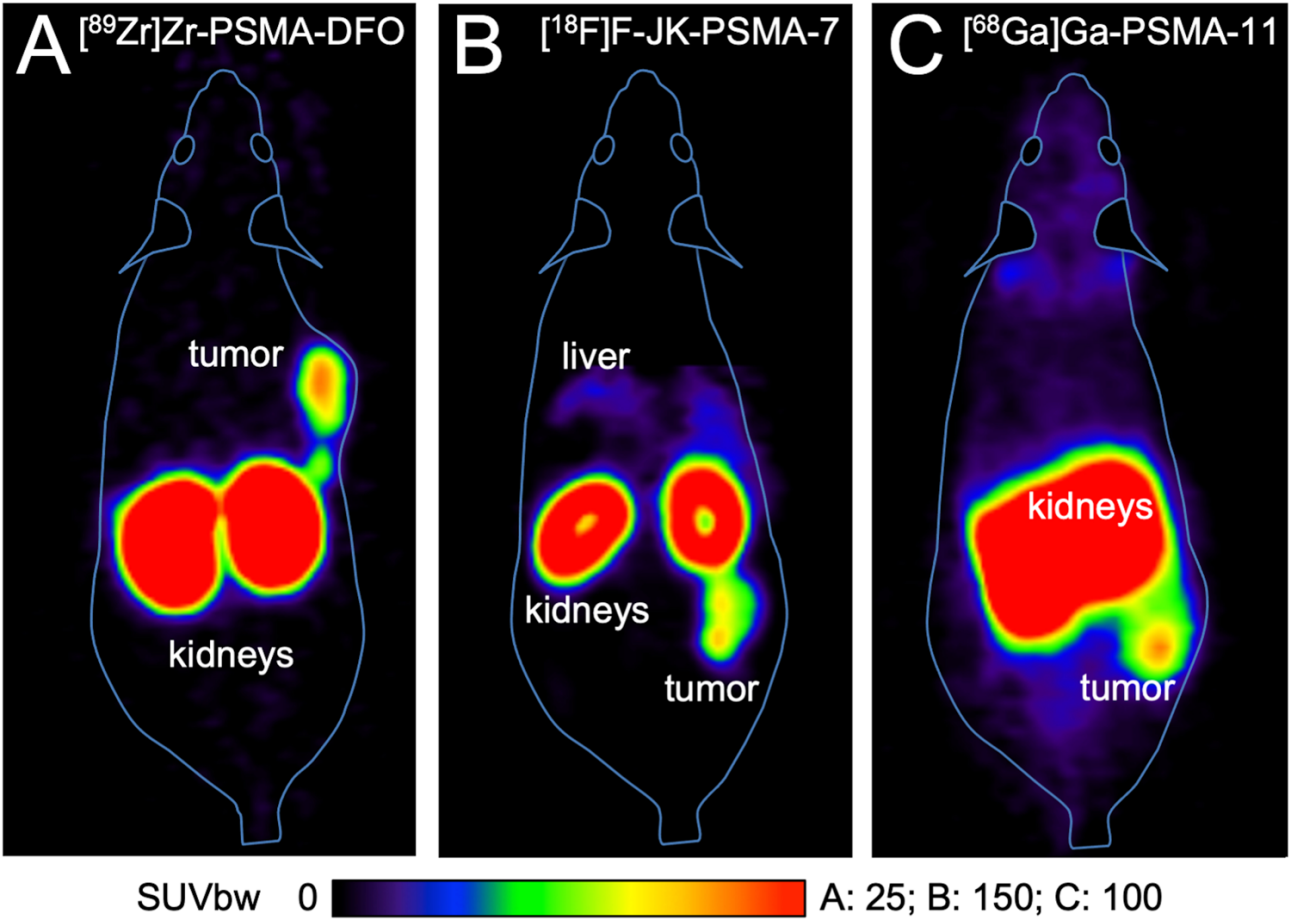
Whole-body horizontal images (sections) of CB17-SCID mice bearing an LNCaP tumor xenograft with [^89^Zr]Zr-PSMA-DFO (**a**), [^68^Ga]Ga-PSMA-11 (**b**), and [^18^F]F-JK-PSMA-7 (**c**), *n* = 1 each. Emission data was acquired 60–120 min p.i.

The LNCaP tumors were clearly visible with all the tracers tested, when measured for 60 min starting at 1 h p.i. (Fig. 2).

**Fig. 3:**
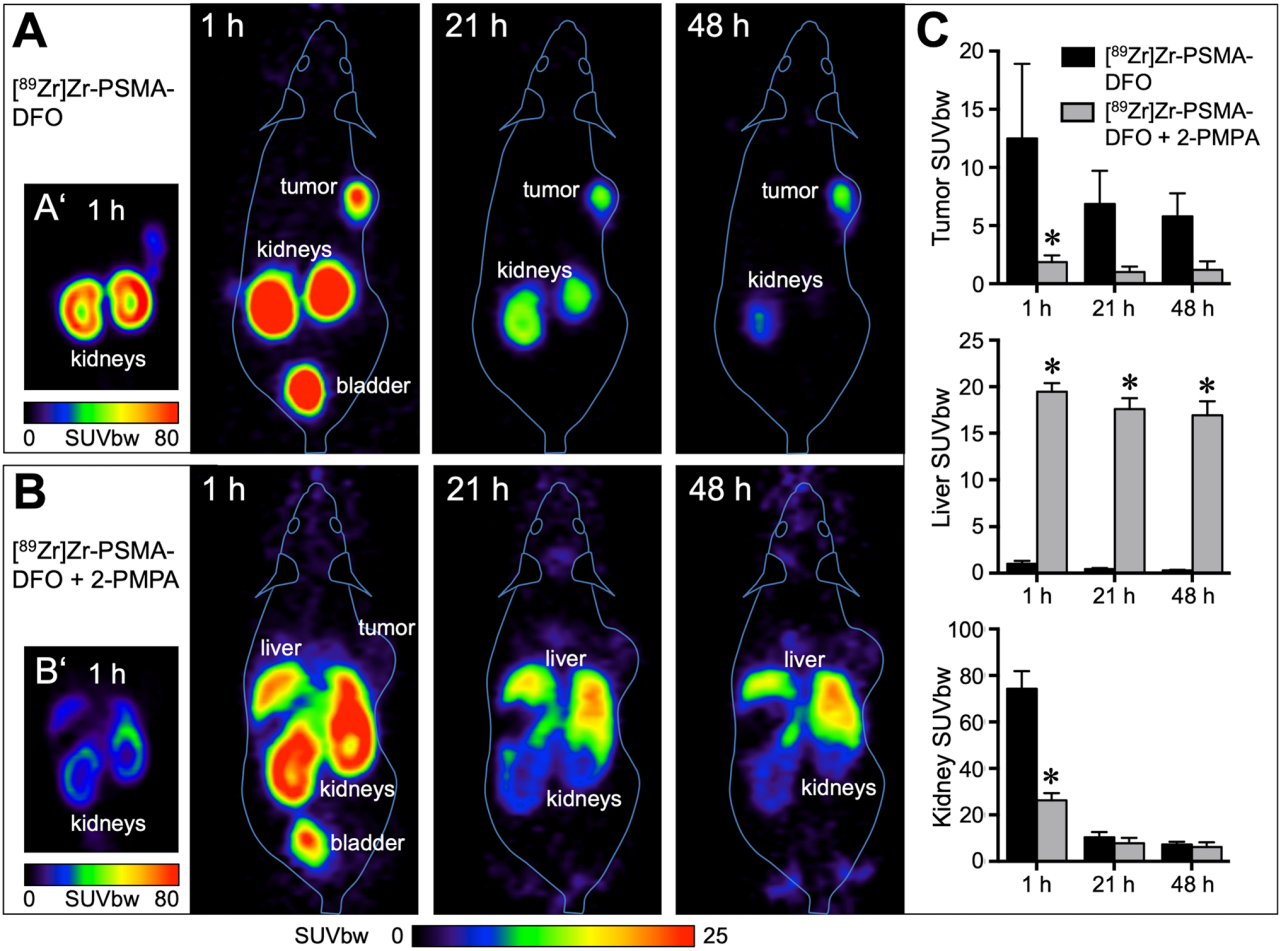
PET imaging of LNCaP tumor xenografts in mice using [^89^Zr]Zr-PSMA-DFO. **a** Representative image from one of three mice measured at three different time points after injection of 10 MBq [^89^Zr]Zr-PSMA-DFO. Even after 48 h, the LNCaP tumor xenograft was still clearly visible. Residual radioactivity was found in the kidneys and the urinary bladder. In A’, kidney radioactivity is shown with a different scaling (SUV_bw_ 0–80 instead of 0–25). **b** Representative image from one of three mice measured at the same time points after injection of 9 MBq [^89^Zr]Zr-PSMA-DFO + 23 mg/kg 2-PMPA. Radioactivity in the tumor was strongly reduced by the addition of 2-PMPA. Instead, radioactivity accumulated in the liver. B’ shows that 2-PMPA reduced radioactivity accumulation in the kidneys as well. **c** Quantitative evaluation with *n* = 3 per group. [^89^Zr]Zr-PSMA-DFO uptake in tumor and kidney was significantly reduced with 2-PMPA after 1 h. Radioactivity accumulation in the liver was significantly increased with 2-PMPA at 1 h, 21 h, and 48 h after injection. All images and values are decay-corrected.

Owing to the longer physical half-life of Zr-89, it was also possible to obtain PET images up to 48 h after injection (*N* = 3, Fig. 3).

The PSMA-inhibitor 2-PMPA markedly reduced the tumor accumulation of the Zr-89-labeled PSMA-targeting vector (*F*(1,4) = 10.3, *p* = 0.0324, post hoc* p* < 0.05 after 1 h). At the same time, the accumulation of radioactivity in the kidneys was also reduced (*F*(1,4) = 67.9, *p* = 0.0012, post hoc* p* < 0.05 after 1 h). Instead, radioactivity accumulated in the liver (*F*(1,4) = 2236, *p* < 0.0001, post hoc* p* < 0.05 after 1 h, 21 h, and 48 h). The clearance of radioactivity from the kidneys was much faster than from the tumor tissue.

### [^89^Zr]Zr-PSMA-DFO in a Patient with PCA Recurrence

**Fig. 4:**
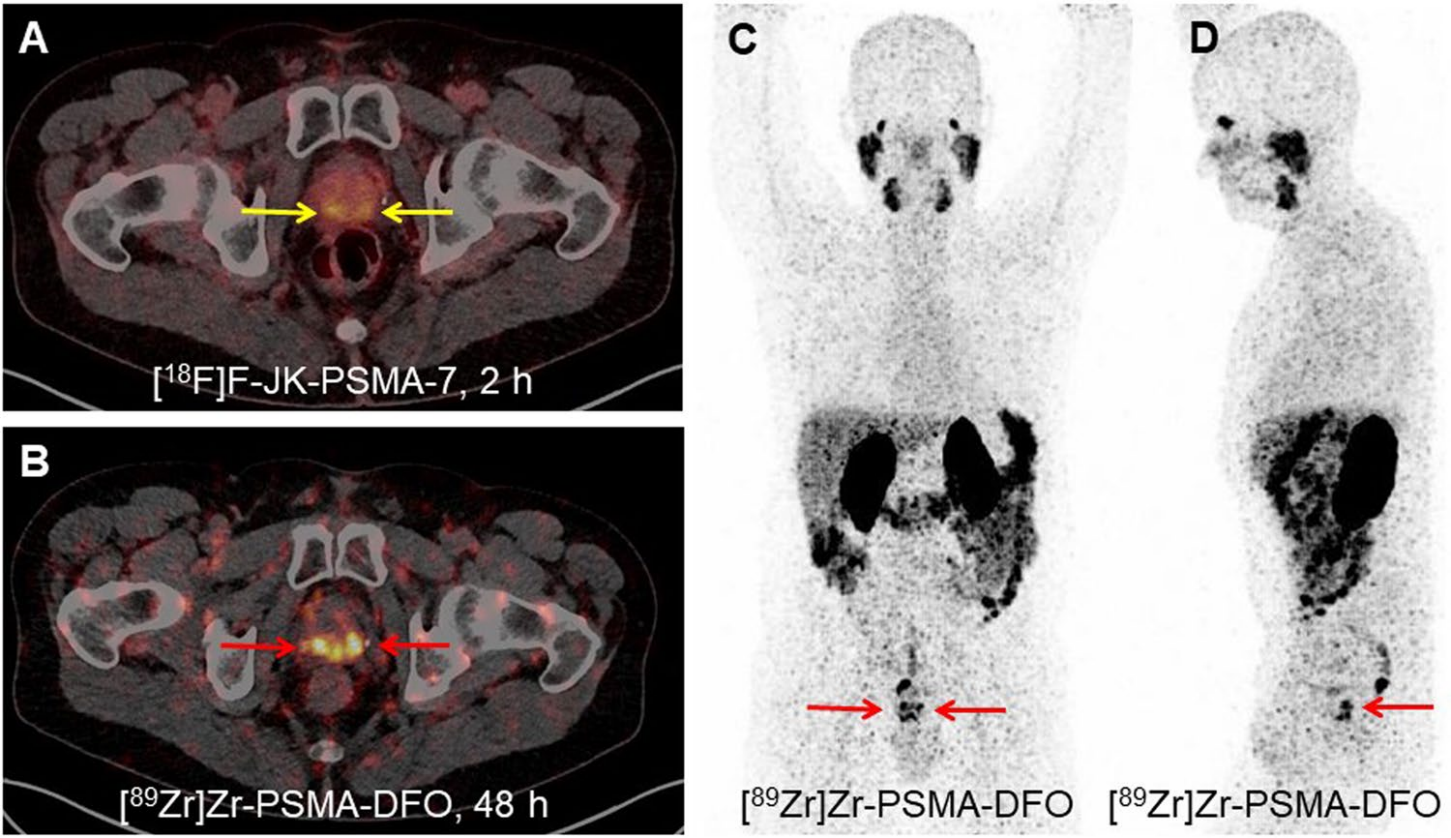
PET/CT imaging of a histologically confirmed relapse of prostate cancer after brachytherapy. Before the first scan, the patient (Gleason score 3 + 4) received 343 MBq [^18^F]F-JK-PSMA-7 and after 6 days 93 MBq [^89^Zr]Zr-PSMA-DFO for the second scan. **a** The [^18^F]F-JK-PSMA-7 PET/CT 2 h p.i. was interpreted as equivocal (SUV_max_ 5.37 in the right prostate lobe, 4.63 in the left prostate lobe, yellow arrows) in conjunction with a previously negative biopsy. **b** The additional [^89^Zr]Zr-PSMA-DFO PET scan 48 h p.i. demonstrated intensive tracer accumulation in the right (SUV_max_ 13.25) and in the left prostate lobe (SUV_max_ 9.47) (red arrows). The SUV_max_ values on the PET scan after 72 h were 13.05 in the right prostate lobe and 7.14 in the left prostate lobe. The repeat biopsy revealed cancer cells on both sides. **c**, **d** The maximum intensity projections (MIP) of the [^89^Zr]Zr-PSMA-DFO PET reveal the PSMA overexpression in the relapse (red arrows) without overlay due to activity in the bladder.

A first-in-human study was conducted with [^89^Zr]Zr-PSMA-DFO in a 60-year-old patient with BCR (Fig. 4). The [^89^Zr]Zr-PSMA-DFO PET scan demonstrated intensive tracer accumulation in the right prostate lobe dorsal (SUV_max_ 13.25, 48 h p.i.) and in the left prostate lobe dorsal (SUV_max_ 9.47). Signal-to-noise ratios (SNR) of [^89^Zr]Zr-PSMA-DFO were 1.9 and 2.0 in the first and second PET scans, respectively. These ratios were lower than those obtained for [^18^F]F-JK-PSMA-7 PET at 5.5. However, [^89^Zr]Zr-PSMA-DFO PET/CT exhibited higher contrast-to-noise ratios (CNR) in PSMA-positive lesions (right prostate lobe 3.8 and 3.0 in scans 1 and 2/left prostate lobe 3.5 and 1.1 in scans 1 and 2), compared with [^18^F]F-JK-PSMA-7 (right prostate lobe 0.9/left prostate lobe 0.6). This suggests that the detection of the two weak PSMA-avid lesions in our patient was facilitated by the higher CNR of [^89^Zr]Zr-PSMA-DFO. The [^89^Zr]Zr-PSMA-DFO PET scans showed the kidneys to be the organ with the highest radiation exposure. We estimated the kidney dose to be 2.8 mGy/MBq. The overall effective dose (ICRP 60) was 0.11 mSv/MBq. The biopsy was repeated in the light of the intensive PSMA overexpression in the prostate lobes shown by [^89^Zr]Zr-PSMA-DFO PET. The second histopathology revealed cancer cells on both sides and the finding with [^89^Zr]Zr-PSMA-DFO PET was confirmed histopathologically.

## Discussion

The following results can be derived from the first radiopharmaceutical, biochemical, and biological data obtained with [^89^Zr]-Zr-PSMA-DFO, a PSMA-targeting agent with a longer half-life than Ga-68- or F-18-PSMA ligands, with which it was compared:

1. [^89^Zr]Zr-PSMA-DFO has been produced with high radiochemical purity of > 99%, stability lasting at least 7 days. Neither the biokinetics nor the examinations using small-animal PET showed a time-dependent increase in Zr-89 accumulation in bone 24 h or 48 h p.i. In view of the known affinity of unchelated zirconium to bone [15, 23, 24], our results indicated a convincing *in vivo* stability.
2. The biochemical and biological properties of [^89^Zr]Zr-PSMA-DFO derived from cellular experiments were comparable to those of [^68^Ga]Ga-PSMA-11 and [^18^F]F-JK-PSMA-7 during the first 5 h.
3. Our preclinical data on [^89^Zr]Zr-PSMA-DFO in LNCaP tumor xenografts in mice demonstrated significantly higher tumor/background ratios after an application interval of 24 h compared with [^68^Ga]Ga-PSMA-11 and [^18^F]F-JK-PSMA-7 after an application interval of 2 h or 4 h.
4. With [^89^Zr]Zr-PSMA-DFO animal PET, the tumor xenograft remained clearly visible over a prolonged period of 48 h.
5. Late PET scans (48 h and 72 h p.i.) in a patient with biochemical relapse indicated a potentially higher contrast-to-noise ratio of [^89^Zr]Zr-PSMA-DFO for localization of tumor areas with a weak PSMA expression on a preceding F-18-labeled PSMA PET.

In the search for a suitable radionuclide for the production of PSMA-targeting ligands, allowing a PET scan to be performed at least 24 h p.i., zirconium-89, which, was considered a promising candidate. The zirconium isotope has become established in immuno-positron emission tomography (PET) imaging in recent years and, with a half-life of 3.27 days, meets the requirements better than Cu-64 (0.53 day), Tb-152 (0.73 day), or Sc-44 (0.167 day)—PET radionuclides whose suitability for the production of radioactive PSMA ligands was demonstrated not so long ago [25–29]. The proven high long-term stability of the Zr-89-labeled, PSMA-affine PET tracer in combination with its long half-life also has practical advantages. There is no need to produce the radiopharmaceutical several times a week. Of course, one could argue that nowadays F-18 tracers can also be easily obtained via existing radiopharmaceutical networks in most areas with PET scanners. Nevertheless, the fact that the [^89^Zr]Zr-PSMA-DFO can be stored for a longer period of time and can be applied 7 days after synthesis with a one-off tracer production run speaks for itself. Apart from that, [^89^Zr]Zr-PSMA-DFO can also be transported over longer distances without any major loss of quality.

The labeling procedure, including cleaning and quality control, was feasible within a time frame of about 120 min. The yield based on the Zr-89 activity employed was 75% in optimum cases. After final purification, the radiochemical purity was excellent and remained > 99% even on storage in aqueous solution for 7 days*.*

The linear relationship between the molar activities of the radioactive PSMA vectors examined in this study and their maximum achievable concentration on 10^6^ tumor cells (*B*_max_) can be summarized as follows: The higher the molar activity of the radioligand, the more radioligand molecules can be bound to a given number of tumor cells. The assumption derived from this that with higher molar activity, there are also higher radioactivity concentrations in the tumor tissue and higher tumor-background ratios have not been confirmed (see the “Limitations” section).

Nevertheless, the key question as set out in the introduction, of whether the results obtained with [^89^Zr]Zr-PSMA-DFO correspond to those obtained with [^68^Ga]Ga-PSMA-11 and [^18^F]F-JK-PSMA-7 or are better, can be answered as follows: [^89^Zr]Zr-PSMA-DFO showed no inferiority compared to the other PSMA tracers regarding *in vitro* experiments for affinity, binding to prostate cancer tumor cells, and biokinetics.

PSMA-DFO, which serves as an *in vivo* vehicle for Zr-89, is itself a relatively small molecule with faster clearance than, for example, an antibody. This is reflected in the low levels of activity, for example in blood and muscles even at 2 h p.i. The question therefore arises whether it makes sense to combine a relatively long-lived nuclide with a ligand with a short biological half-life. Our cell biological studies showed that [^89^Zr]Zr-PSMA-DFO showed significant internalization. In the context of this work, it can only be assumed that this leads to a re-complexation of the zirconium within the cell and a binding to intracellular molecules. This assumption is supported by a number of studies. Current publications show that Zr-89 is bound intracellularly after it has been able to penetrate the cell wall as a lipophilic complex [30, 31]. Fung et al.[32] observed differences in the clearance rates of radioactivity from the tumor for two forms of the humanized monoclonal antibody J591 ([^124^I]I-J591 and [^89^Zr]Zr-J591) against prostate-specific membrane antigen (PSMA) in mice with LNCaP tumors. So the only difference between the radioimmunoconjugates was the type of radiolabeling. The authors attribute this difference to typical zirconium trapping mechanisms within the tumor cells which do not operate in the case of non-residualizing of iodine. Similar results were found by Cheal et al. [33] when comparing an antibody against the clear cell renal carcinoma labeled with Zr-89 or I-124. These and other authors conclude that Zr-89 is a residualizing isotope and remains in cells after internalization, allowing activity to accumulate and concentrate in tumor cells while removing non-localized activity from the body, ultimately resulting in high-contrast images.

Our blocking experiment with 2-PMPA showed that in the mouse approximately two-thirds of renal radioactivity is due to specific binding of the PSMA tracer. Around one-third is not blockable, and therefore reflects renal excretion of the radiotracer and its metabolites. Forty-eight hours after injection of [^89^Zr]Zr-PSMA-DFO, there was no detectable radioactivity in the bladder in mice. Thus, Zr-89 is well-suited to detect lesions in the genitourinary tract at late time points, when radioactivity has already cleared from the kidneys and bladder. The scheduling of tracer injection and PET scans on different days was chosen as the optimal arrangement for a few patients with a rare constellation of findings and on account of patient preference.

On the basis of the excellent preclinical imaging properties of [^89^Zr]Zr-PSMA-DFO, we carried out the first observational study on [^89^Zr]Zr-PSMA-DFO PET/CT in a patient with BCR. The activity used (93 MBq) was not derived from a phase-1 trial, but was analogous to the labeling of antibodies with Zr-89 [34]. The first use in humans resulted in a promising performance with regard to clear localization of PSMA-positive tumor tissue when the preceding PET with [^18^F]F-JK-PSMA-7 had been interpreted as PSMA-negative or equivocal. The radiation exposure should be weighed against the potential benefit of metastasis-directed therapy or salvage radiotherapy. Additional clinical data in a series of patients will be published in due course and will evaluate whether [^89^Zr]Zr-PSMA-DFO PET can improve the contrast-to-noise ratio in patients with weakly PSMA-positive lesions.

### Limitations

Some of the tumor/background ratios determined by measuring the radioactivity of organ samples are unexpected. The highest tumor-muscle ratio was determined just 2 h after the injection of [^89^Zr]Zr-PSMA-DFO. The tumor-to-background ratios for [^68^Ga]Ga-PSMA-11 were significantly lower compared to those of the other radioactive PSMA vectors. It is noticeable that, for example, the tumor/blood ratios given in the literature for [^68^Ga]Ga-PSMA-11 at 2 h p.i. differ widely between individual studies [34–37]. In animal experiments on mice, it should be taken into account that owing to the low blood volume, and variations in the molar activities of the radioactive PSMA ligand used by different authors, different tumor models, or mouse strains can produce different results.

Our work should be seen as a first feasibility study to investigate the suitability of [^89^Zr]Zr-PSMA-DFO for PET examinations of prostate carcinoma lesions with weak PSMA expression. The data collected are not yet sufficient to make generalizable statements about radiation exposure from the radiotracer. This is the subject of a study involving several BCR patients that will be published soon.

## Conclusion

This preclinical study demonstrates that the tumor uptake and biodistribution of [^89^Zr]Zr-PSMA-DFO in normal tissues is comparable to that of [^68^Ga]Ga-PSMA-11 and [^18^F]F-JK-PSMA-7 in the first 5 h. After an application interval of 24 h, significantly higher tumor/background ratios could be achieved with [^89^Zr]Zr-PSMA-DFO than with [^68^Ga]Ga-PSMA-11 and [^18^F]F-JK-PSMA-7 at 2 h and 4 h after application. The first-in-human application in a patient with BCR indicated the potential advantage of [^89^Zr]Zr-PSMA-DFO in the localization of tumors with low PSMA expression on an F-18-labeled PSMA PET. Thus, [^89^Zr]Zr-PSMA-DFO represents a useful addition to the set of PET radiopharmaceutical instruments available for the detection of prostate carcinoma lesions. Clinical investigations of suitable patients with a diagnostic gap in the localization of a biochemical relapse would therefore be worth pursuing.

## Supplementary Information

Below is the link to the electronic supplementary material.

Supplementary file1 (DOCX 168 kb)

Supplementary file2 (DOCX 595 kb)

Supplementary file3 (DOCX 14 kb)
