# Supplementary material for: Translational Development of a Zr-89-Labeled Inhibitor of Prostate-specific Membrane Antigen for PET Imaging in Prostate Cancer
Source: Mol Imaging Biol. 2021 Aug 9;24(1):115–25. doi: 10.1007/s11307-021-01632-x (PMC8760230; doi:10.1007/s11307-021-01632-x)
Supplement: Supplementary file 1 — Supplementary file1 (DOCX 168 kb) [file 11307_2021_1632_MOESM1_ESM.docx]

Supplemental data

Table 1: Specific binding of radiolabeled ligands in LNCaP cells as percentage of total activity (n=3)

| Time | [^89^Zr]Zr-PSMA-DFO | [^68^Ga]Ga-PSMA-11 | [^18^F]F-JK-PSMA-7 |
| --- | --- | --- | --- |
| 0.5 h | 29.1±2.1 | 27.7±0.7 | 23.8±0.6** |
| 1 h | 38.5±2.3 | 35.3±3.1* | 32.6±0.4** |
| 2 h | 46.5±1.3 | 42.0±1.2* | 38.8±1.3** |
| 3 h | 48.8±1.0 | 46.3±2.1 | 45.2±1.9* |
| 5 h | 49.4±1.3 | 48.3±1.2 | 45.8±0.4* |

*, ** significantly lower compared to [^89^Zr]Zr-PSMA-DFO (*p<0.05; **p<0.001)

Table 2. Specific internalized activity in LNCaP cells expressed as a percentage of cellular activity relative to specific cell-bound activity (n=3).

| Time | [^89^Zr]Zr-PSMA-DFO | [^68^Ga]Ga-PSMA-11 | [^18^F]F-JK-PSMA-7 |
| --- | --- | --- | --- |
| 0.5 h | 32.7±4.1 | 28.9±0.7 | 19.6±1.3** |
| 1 h | 39.8±0.4 | 39.7±2.0 | 33.9±1.6* |
| 2 h | 48.0±3.4 | 48.3±2.5 | 44.3±2.5 |
| 3 h | 50.0±0.6 | 50.5±3.4 | 46.0±2.2 |
| 5 h | 61.7±3.4 | 56.8±2.2* | 48.6±0.9** |

*, ** significantly lower compared to [^89^Zr]Zr-PSMA-DFO (*p<0.05; **p<0.001)
