# Supplementary material for: Translational Development of a Zr-89-Labeled Inhibitor of Prostate-specific Membrane Antigen for PET Imaging in Prostate Cancer
Source: Mol Imaging Biol. 2021 Aug 9;24(1):115–25. doi: 10.1007/s11307-021-01632-x (PMC8760230; doi:10.1007/s11307-021-01632-x)
Supplement: Supplementary file 2 — Supplementary file2 (DOCX 595 kb) [file 11307_2021_1632_MOESM2_ESM.docx]

**Incubation for 30 min at 35 ^o^C**

296 µl 25 µM EDTA solution

(pH 4.3 – 4.5) +

74 µl of precursor (I) (1 nmol/ µl DMSO)


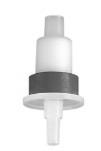


1.Water

Waste Water

[Fe(III)EDTA]^-^

EDTA

2. EtOH

MidiTrap

G-10

MWCO: 700


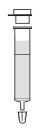


Solvent exchange

Waste: EtOH

Water

EuK-2NaI-Amc-N-sucDf (II, iron-free precursor)

in aqueous solution

Zr-89 in 1 M oxalic acid

1 M Na_2_CO_3_

0.5 M HEPES

0.25 M NaOAc


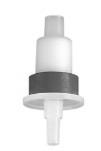


Water

[^89^Zr]Zr-PSMA-DFO

Saline

solution

Waste Water

Free Zr-89

70% EtOH

**I: EuK-2Nal-AMCH-N-sucDf-Fe**

**Precursor**

**II: EuK-2Nal-AMCHN-sucDf**

**Precursor (iron-free)**

**III: [^89^Zr]Zr-PSMA-DFO**

**Radioligand**

II (iron free precursor)

Supplementary Figure 1 Schematic demonstration of labeling procedure of.

Step 1 is the remove of Fe (III) from EuK-2Nal-AMCH-N-sucDf-Fe by transchelation to EDTA. 100 µg (74 nmol) of precursor is dissolved in DMSO (1 nmol/µl). To this solution, 296 µl (7.4 µmol) of an EDTA solution (25 µM) is added and the reaction mixture is incubated for 30 min at 35 ^o^C (pH 4.3 – 4.5). Step 2 is the isolation of the free iron compound. After 30 min, EDTA and [Fe(III)EDTA]^-^ are removed by solid phase extraction (Sep-Pak™ C_18_ plus light cartridge). Then the free iron compound in EtOH is passed through a PD MidiTrap™ G-10 column to have it in water (1.2 ml). Step 3 is the labeling EuK-2Nal-AMCH-N-sucDf. To 1 ml of Zr-89 in 1 M oxalic acid solution, 1.2 ml of 1 M sodium carbonate, 2 ml of 0.5 M HEPES (pH 6.8 - 7.2) are added, followed by 838 µl of 0.25 M sodium acetate (gentisic acid 5mg/ml) and 162 µl of the free iron compound in water, final pH 6.8-7.2. After 2 h, reaction mixture mixture (5.2 ml) is given to a Sep-Pak™ C_18_ plus light cartridge and the Zr-89-N-*suc*Df-AMCHA-2Nal-EuK ([^89^Zr]Zr-PSMA-DFO) is eluted with 1 ml of 70% EtOH.
