# Supplementary material for: Translational Development of a Zr-89-Labeled Inhibitor of Prostate-specific Membrane Antigen for PET Imaging in Prostate Cancer
Source: Mol Imaging Biol. 2021 Aug 9;24(1):115–25. doi: 10.1007/s11307-021-01632-x (PMC8760230; doi:10.1007/s11307-021-01632-x)
Supplement: Supplementary file 3 — Supplementary file3 (DOCX 14 kb) [file 11307_2021_1632_MOESM3_ESM.docx]

K_d_ = 5.07 ± 0.45 nM

[^18^F]F-JK-PSMA-7

K_d_ = 5.15 ± 0.60 nM

[^68^Ga]Ga-PSMA-11

[^89^Zr]Zr-PSMA DFO

K_d_ = 4.97 ± 0.57 nM

Supplementary Figure 2.

Radioligand binding assay on LNCaP cells. The total, specific and non-specific binding has been plotted against increasing concentrations ranging from 0.25 to 75 nM of [^89^Zr]Zr-PSMA-DFO, [^68^Ga]Ga-PSMA-11 and [^18^F]F-JK-PSMA-7
